# A Low-cost, Highly-stable Surface Enhanced Raman Scattering Substrate by Si Nanowire Arrays Decorated with Au Nanoparticles and Au Backplate

**DOI:** 10.1038/s41598-017-04062-4

**Published:** 2017-07-04

**Authors:** Bi-Shen Lee, Ding-Zheng Lin, Ta-Jen Yen

**Affiliations:** 1 Department of Material Science and Engineering, National Tsing Hua University, Hsinchu, 30013 Taiwan; 2 Department of Material and Chemical Research Laboratories, Industrial technology and research institute (ITRI), Hsinchu, Taiwan

## Abstract

We present a facile and cost-effective manner to fabricate a highly sensitive and stable surface enhanced Raman scattering (SERS) substrate. First, a silicon nanowire array (SiNWA) is tailored by metal-assisted chemical etching (MaCE) method as a scaffold of the desired SERS substrate. Next, with an oblique angle deposition (OAD) method, optimized gold nanoparticles (AuNPs) are successfully decorated on the surface of the SiNWA. These AuNPs enable a strong localized electric field, providing abundant hot spots to intensify the Raman signals from the targeting molecules. By applying a well-established methodology, Taguchi method, which is invented for designing experiments, the optimized combination of parameters is obtained efficiently. The experimental results are also confirmed by finite-difference time-domain (FDTD) simulation calculations. Besides, a gold metal backplate (AuMBP) is applied to further enhancing the Raman signal intensity. Based on this developed SERS substrate, we demonstrated an enhancement factor (EF) of 1.78 × 10^6^ and a coefficient of variation (CV) of 4.2%. Both EF and CV indicate a highly stable property and the optimized SERS substrate substantially outperform the commercial product. In the end, we also demonstrate a quantitative measurement on practical application of detecting malachite green (MG) with concentration from 10 nM to 100 μM.

## Introduction

Food safety is a serious public issue because of illegal food additives. As a result, an urgent demand of developing a rapid detection method with a good sensitivity appears. To date the typical analytical techniques such as high-performance liquid chromatography (HPLC)^1^, liquid chromatography/mass spectroscopy (LC/MS)^2^ and immune-assays^3, 4^, are time-consuming and pricy. Also they are designed for specific analysis as the solution for fast screening and trace detection method. As a consequence, Raman scattering technique is regarded as a promising candidate for practical applications because it allows label-free process to reveals the fingerprint spectra of targeting molecules. More importantly, comparing to conventional infrared absorption techniques, the Raman signal is free from water interference and thus make it suitable for wet samples^5^. However, the scattering cross section of Raman signals is typically small. So the scientists require to substantially enhancing the Raman signals. In 1974, a signal-enhancement phenomenon called surface enhanced Raman scattering (SERS) was discovered by Fleischmann *et al*. as collecting the Raman signal of pyridine molecules adsorbed at a silver electrode^6^. By means of the SERS technique, the conventional Raman signal can be enormously intensified to enable single molecule detection^7^.

The SERS effect dominantly stems from the strong electromagnetic enhancement that is typically provided by exciting plasmonic resonances in the metallic structures^8^. Generally, close to the roughened or nanostructured metal surface, the local electromagnetic field can be efficiently magnified^9^, so noble metal nanoparticles have been applied as SERS substrates^10, 11^. Recently, scientist demonstrated the detection of a variety of toxic molecules including melamine^12^, malachite green^13^, ractopamine^14^, and also DNA and RNA recognition^10, 15^. To further improve the SERS effect, gold quasi-3D array^16^, ordered nanoporous AuNP array^17^, gold nanofingers^18^, and especially the glass nanopillar array covered with silver nanoislands^19^ were proposed. The last case shows an average enhancement factor near 10^7^. Nevertheless, the glass nanopillar array involves expensive reactive ion etching (RIE) fabrication process and the silver is not chemically stable as gold. Thus, these two issues impede its employment for fast screening and practical applications. Herein, we reported a facile and low-cost and sensitive SERS substrate for substitution. At first, we utilized a metal-assisted chemical etching (MaCE) method to fabricate Si nanowire arrays (SiNWA)^20, 21^, which function as a high surface-to-volume scaffold. Then, the scaffold was deliberately deposited AuNPs by oblique angle deposition to yield strong localized surface plasmon resonance for enhancing the Raman signal. Similar OAD method to form gold or silver tailored structure for SERS was demonstrated previously^22–24^. Finally, an Au metal backplate (AuMBP) was also introduced to preventing the light trapping by SiNWA^25^, leading to a further enhancement of our engineered SERS substrate.

## Materials and Methods

### Fabrication of SiNWA via MaCE

**Figure 1:**
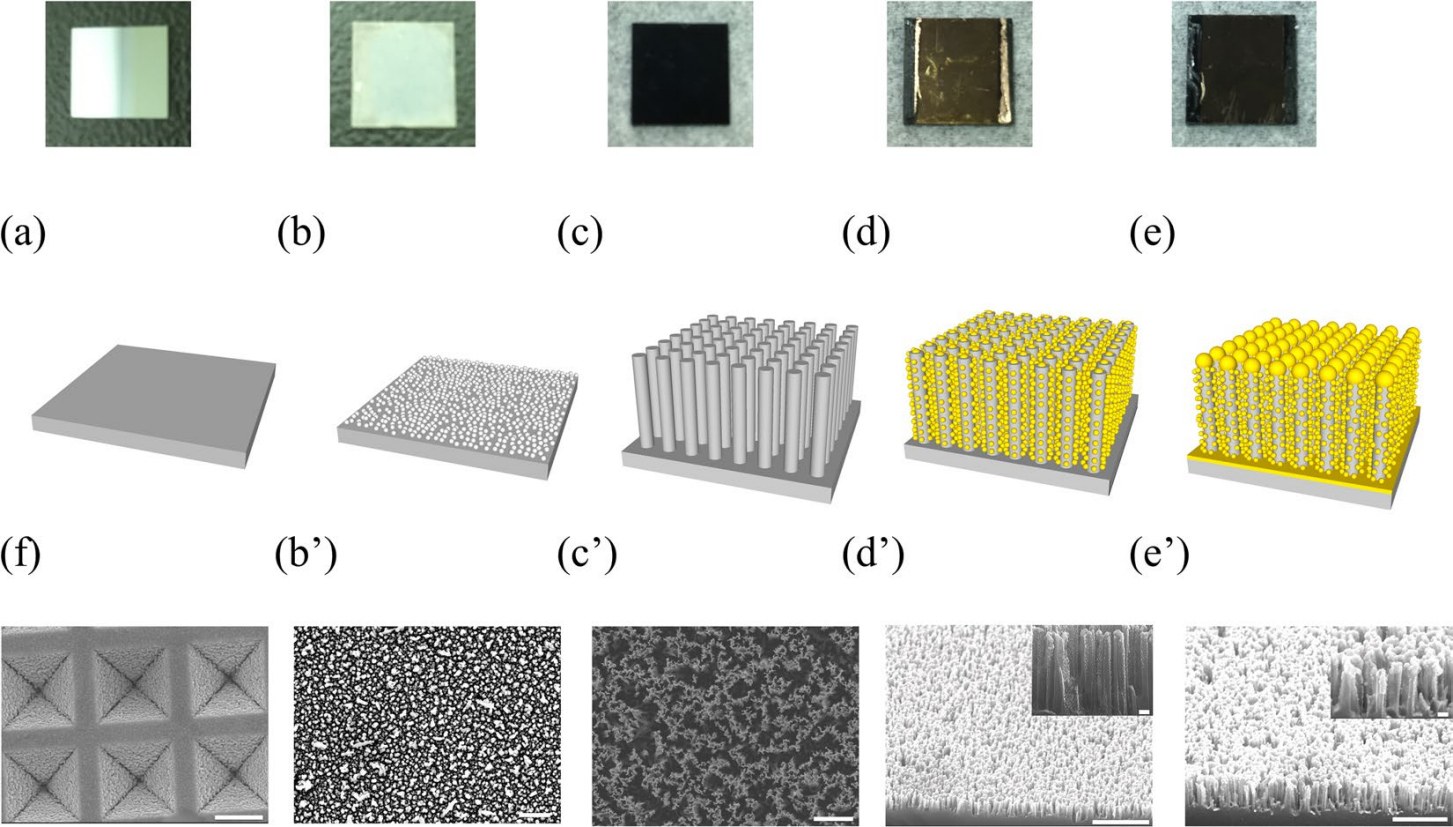
Schematic diagram of experimental process flow (**a**) The cut <100> P-type 1 × 1 cm^2^ silicon substrate. (**b**) Silicon substrate with silver network as the catalyst for the etching process. The blue region is represented for Ag clusters. (**c**) The SiNWA scaffold for the desired SERS substrate. (**d**) The SiNWA with AuNPs decorated on sidewall by OAD process. (**e**) The SiNWA with AuNPs and AuMBP as the desired SERS substrate. (**f**) SEM figure of commercialized SERS substrate (Klarite). It is noted that Figure (**b**’), (**c**’), (**d**’), (**e**’) are the SEM figures corresponded to each steps. (The scale bar represented 1 μm in the lower magnitude SEM figure and represented 100 nm in the inset higher magnitude figure).

A boron-doped (p-type) silicon (100) (1–10 Ω-cm) wafer was precisely cut into small chips in 1 × 1 cm^2^ for the SERS substrates as shown in Fig. 1(a). Second, to fabricate the SiNWA, we herein applied a wet chemical etching method termed MaCE, which was an anisotropic etching method for silicon^20, 21, 26^. In the wet etching process, the electrolyte, consisting of 4.6 M hydrogen fluoride (HF) and 0.44 M silver nitrate (AgNO_3_) was prepared at room temperature. The as-prepared silicon chips were dipped into the electrolyte mixture solution for 10 s. After this step, the silicon surfaces were deposited with silver networks because of the exchange of electrons between the silver ions in AgNO_3_ and silicon atoms, as shown in Fig. 1(b) and the corresponding SEM Fig. 1(b’). Then, the silicon chips with silver networks were immersed into another etching solution composed of HF and H_2_O_2_ with concentration equal to 4.6 M and 0.44 M, respectively. The etching processes were all conducted in this step under room temperature. It is noted that the depths of SiNWA can be well controlled by the etching time. The entire galvanic formulation is listed below^27^:

$${\rm{Cathode}}:{{\rm{H}}}_{2}{{\rm{O}}}_{2}+2{{\rm{H}}}^{+}+2{{\rm{e}}}^{-}\to 2{{\rm{H}}}_{2}{\rm{O}}$$

$${\rm{Anode}}:{\rm{Si}}+2{{\rm{H}}}_{2}{\rm{O}}\to {{\rm{SiO}}}_{2}+4{{\rm{H}}}^{+}+4{{\rm{e}}}^{-};$$

$${{\rm{SiO}}}_{2}+6{\rm{HF}}\to {{\rm{H}}}_{2}{{\rm{SiF}}}_{6}+2{{\rm{H}}}_{2}{\rm{O}};$$

$${\rm{Si}}+6{\rm{HF}}\to {{\rm{H}}}_{2}{{\rm{SiF}}}_{6}+4{{\rm{H}}}^{+}+4{{\rm{e}}}^{-}$$

$${\rm{Overall}}:{\rm{Si}}+2{{\rm{H}}}_{2}{{\rm{O}}}_{2}+6{\rm{HF}}\to {{\rm{H}}}_{2}{{\rm{SiF}}}_{6}+4{{\rm{H}}}_{2}{\rm{O}}$$

After the etching step, the residual Ag networks were removed with a concentrated (wt. 65%) nitric acid solution and the SiNWA was shown in Fig. 1(c) and the top-view SEM figure as shown in Fig. 1(c’).

### Formation of AuNPs and AuMBP onto the SiNWA scaffold

The prepared SiNWA with desired depths was put into the electron-beam evaporator to form AuNPs served as the SERS activated metal. In this step, by applying oblique angle deposition (OAD), the SiNWA were covered with tiny AuNPs as shown in Fig. 1(d) and tilt-view SEM figure shown as Fig. 1(d’). In order to prevent SiNWA from absorbing the light and reduce the scattering intensity, we then deposited a gold metal backplate with desired thickness under normal incidence deposition. After this step, the bottom of SiNWA was covered by a gold layer termed as AuMBP. This additional AuMBP can also reflect the back-scattered field. Finally, the morphology of AuNPs and AuMBP decorated SiNWA is shown as Fig. 1(e) and the corresponding SEM figure as Fig. 1(e’). In addition, we set a commercialized SERS substrate, Klarite, as a benchmark and as a comparison group. The SEM image of Klarite is shown in Fig. 1(f). Scanning electron microscopy (SEM, JSM 6500 F, JOEL) was used to analyze the morphology of desired SERS substrate.

### Design of experiment by Taguchi method

In order to fabricate the desired SERS substrates with good productivity and stable quality, we adopted Taguchi method, a well-established methodology for designing experiments. Firstly, we designed a Taguchi L9 orthogonal array, dealing with four critical parameters in the experimental process: (A) Depths of SiNWA, (B) OAD angle, (c) Thickness of AuNPs, (D) Thickness of AuMBP and each parameter can have three different levels as shown in Table 1.

**Table 1:** The Taguchi L9 orthogonal table with four experimental parameters: (A) Depths of SiNWA, (B) OAD angle, (c) Thickness of AuNPs, (D) Thickness of AuMBP and the corresponding Raman signal intensity for each trial.

Trial No.	Control factor level				Average signal Intensity sited at 1073 cm^−1^ (a.u.)
	A	B	C	D	
1	1	1	1	1	231.44
2	1	2	2	2	656.89
3	1	3	3	3	913.88
4	2	1	2	3	704.27
5	2	2	3	1	580.47
6	2	3	1	2	905.77
7	3	1	3	2	878.70
8	3	2	1	3	934.48
9	3	3	2	1	358.95
**Control factor**	**Label**	**Level 1**		**Level 2**	**Level 3**
Depths of SiNWA (nm)	A	150		300	600
OAD angle (degree)	B	60		70	80
Thickness of AuNPs (nm)	C	10		20	30
Thickness of AuMBP (nm)	D	0		10	20

### SERS spectra measurement

To prepare for Raman measurement, the self-assembled monolayer (S.A.M) thiophenol molecule was set as a Raman reporter. The as-fabricated SERS substrates and the commercialized one (Klarite) were immersed in freshly prepared 10^−2^ M ethanolic thiophenol solution for eight hours to ensure the specific binding of thiophenol molecules on the SERS substrate. Following the reaction period, samples were removed from ethanolic thiophenol solution, copiously rinsed in ethanol, and dried in nitrogen gas to remove unreacted thiophenol molecules and other solvent. In this process, a self-assembled monolayer of thiophenol was formed on the gold surface via S-Au bonds. In Raman measurement, the characteristic peaks of thiophenol molecule are sited at 999, 1024, 1073 cm^−1^ which represent the in-plane ring-breathing mode, in-plane C-H bend, and in-plane ring-breathing mode coupled with the C-S stretching mode, respectively. Here, we chose the most intensive peak sited at 1073 cm^−1^ to compare the performance between different kinds of SERS substrates.

**Figure 2:**
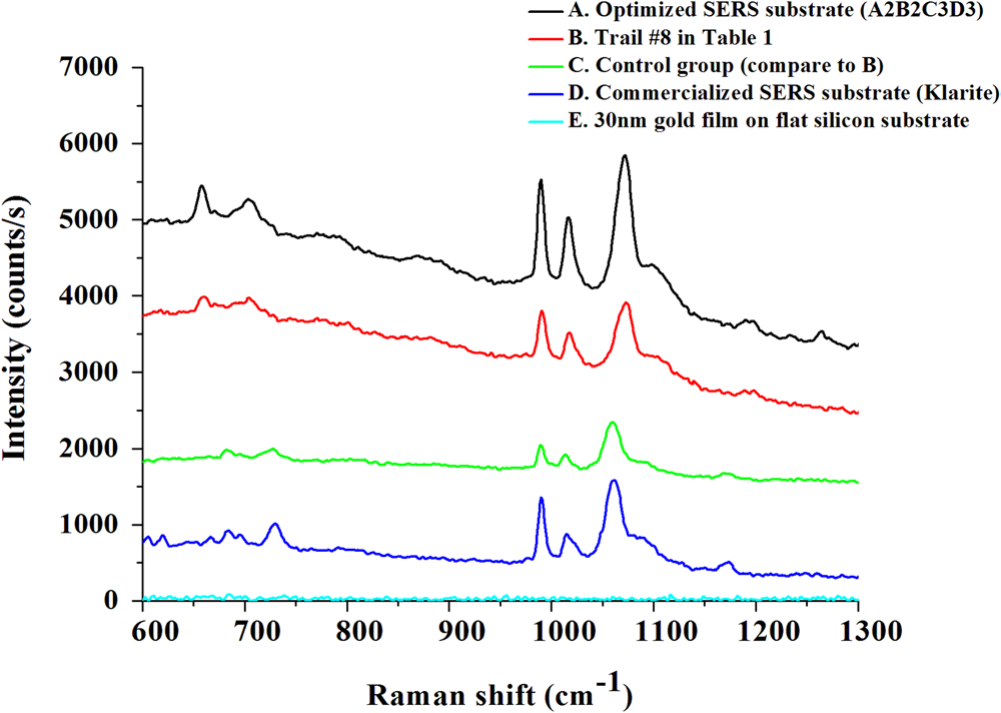
Raman spectrum of 10^−2^ M S.A.M. thiophenol molecules measured from different kinds of SERS substrates (labeled with different colors in the figure). All measurement are conducted under the same laser and surrounding conditions.

For the setup of Raman measurement, a 785 nm near infrared Laser with power equals to 200 mW was utilized to excite the SERS substrate. The Laser source was focused with a 20X objective lens, resulting in a focal spot diameter of 100 μm. Therefore, an illuminating intensity was about 2.54 kW/cm^2^ and the laser integration time was set as 1 second for 10 times average. It is noted that the scattered radiation was collected in a backward direction with the same objective lens. Finally, as shown in Fig. 2, the Raman spectrums of thiophenol molecules measured from different kinds of developed SERS substrates. The average intensity of peak sited at 1073 cm^−1^ and coefficient of variance (C.V.) were also listed in Table 2.

**Table 2:** The average intensity of peak sited at 1073 cm^−1^ and coefficient of variance (C.V.) measured from different kinds of SERS substrates listed in Fig. 2. (The coefficient of variance (C.V.) is calculated by randomly choose five points.

Substrate	A Optimized SERS substrate (A2B2C3D3)	B Trial #8 in Table 1	C Control group (Compare to substrate B)	D Commercialized SERS substrate (Klarite)	E 30 nm gold film on flat silicon substrate
Substrate Composition	300 nm SiNWA 70° OAD 30 nm AuNPs 20 nm AuMBP	600 nm SiNWA 70° OAD 10 nm AuNPs 20 nm AuMBP	600 nm SiNWA 70° OAD 10 nm AuNPs	Au film on textured silicon	30 nm Au film on flat silicon
Average Intensity (counts/s)	1739.5	833.61	573.02	974.5	w/o
Coefficient of Variance	4.2%	5.7%	9.9%	4.2%	w/o

## Results

### The optimized parameters combination by Taguchi method

At first, in the experimental design of Taguchi analysis, the desired result was set as the greater Raman signal intensity the better. The average Raman signals in each trial was measured by randomly chosen five points on the developed SERS substrate, and the corresponding results were listed in Table 1. Based on the measured Raman intensities of nine trials, we conducted an analysis of variance (ANOVA) to identify the significance of four individual parameters, and then concluded the optimal combination of A_2_B_2_C_3_D_3_, which denoted 300 nm-long SiNWA with 70° OAD, 30 nm-thick AuNPs and 20 nm-thick AuMBP, respectively (Refer to Supplementary Material, Table S1 and Figure S1). In addition, to assure the accuracy of the concluded optimal combination, a confirmatory experiment is a must. Therefore, we further fabricated a SERS substrate of A_2_B_2_C_3_D_3_ accordingly. This optimized SERS substrate provided the greatest signal intensity among all our SERS substrates, including nine trials in Table 1 and three other SERS substrates shown in Table 2. Such a confirmatory result substantially credits the optimization process.

### The function of AuMBP

**Figure 3:**
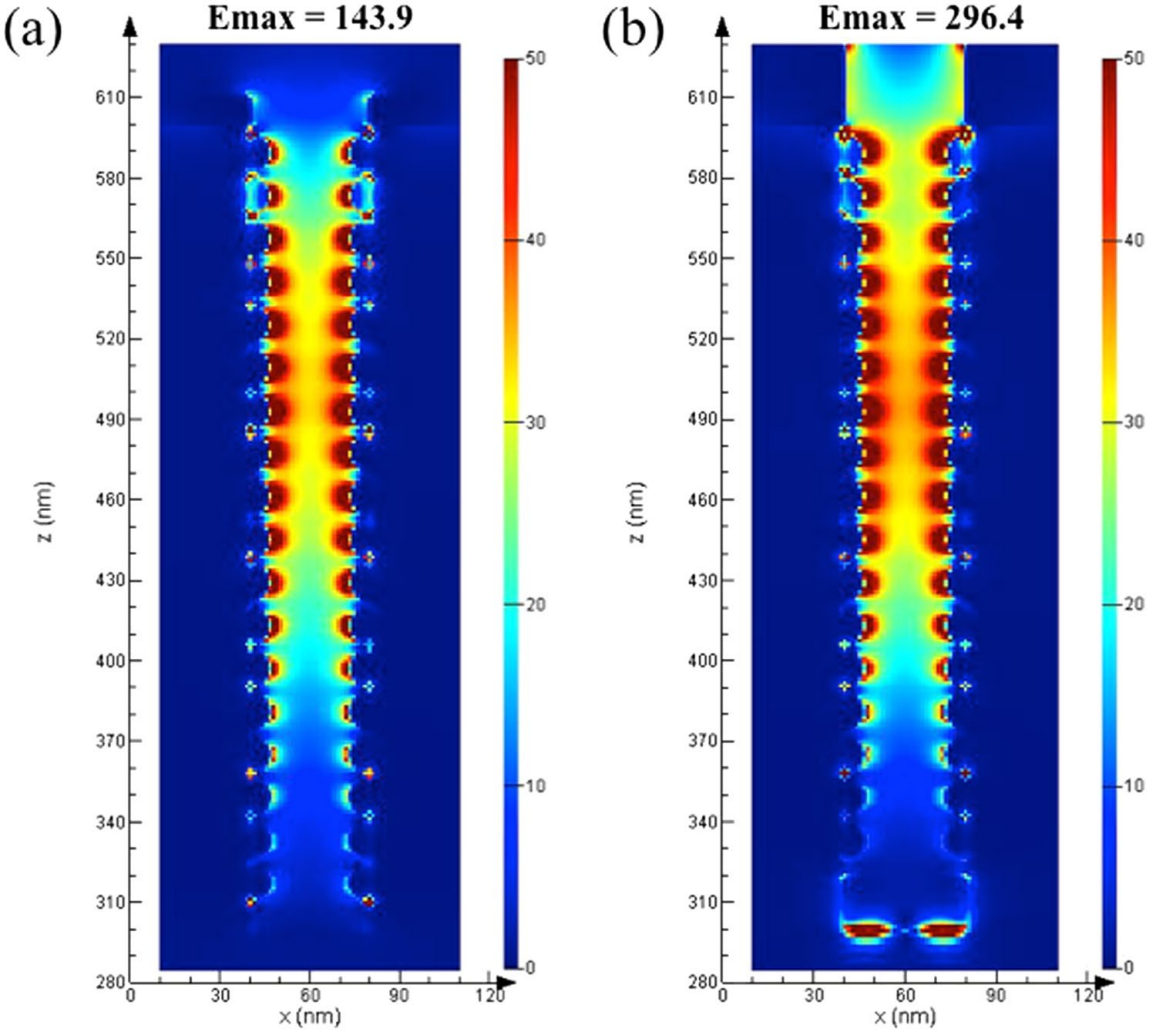
FDTD simulation results of the electrical field intensity for (**a**) Control group (SERS substrate composed by SiNWA with AuNPs). (**b**) Trail #8 SERS substrate (SERS substrate composed by SiNWA with AuNPs and AuMBP). (Note that the diameter of AuNPs on the sidewall is set as 13 nm, the distance between SiNWA is set as 40 nm, and the thickness of AuMBP at the bottom is set as 20 nm).

Next, herein we applied a layer of Au metal back plate (AuMBP) to our SERS substrates to prevent the Raman signals from being trapped by the SiNWA. For comparison, we chose the best result among nine trials in the Taguchi orthogonal array (trial #8, red line), and removed AuMBP as a control (blue line, trial #8 without AuMBP). As shown in Fig. 2, with the AuMBP, the average signal intensity peaked at 1073 cm^−1^ increased 45.4%, from 573 to 833 counts/s, under the same measurement conditions. In addition, we also numerically verified the function of AuMBP with the 3D finite-difference time-domain (3D-FDTD) simulation (*Lumerical Solutions*, *Inc*.). The direction of incident laser beam was set along z-axis and the polarization direction was along x-axis and the boundary condition of x and y directions are set as perfect matching layer (PML). As shown in Fig. 3, it is proved with the AuMBP, the intensity of maximum localized electric field became twice greater because of the much stronger backscattering from the gold backplate. Consequently, the stronger reflectance facilitates additional plasmonic resonance at the AuNPs on the sidewall of the SiNWA, intensifying the amounts of hot spots under 785 nm laser excitation that agrees with the measurement results well as shown in Fig. 2.

## Discussion

### Quantitative measurement of malachite green

**Figure 4:**
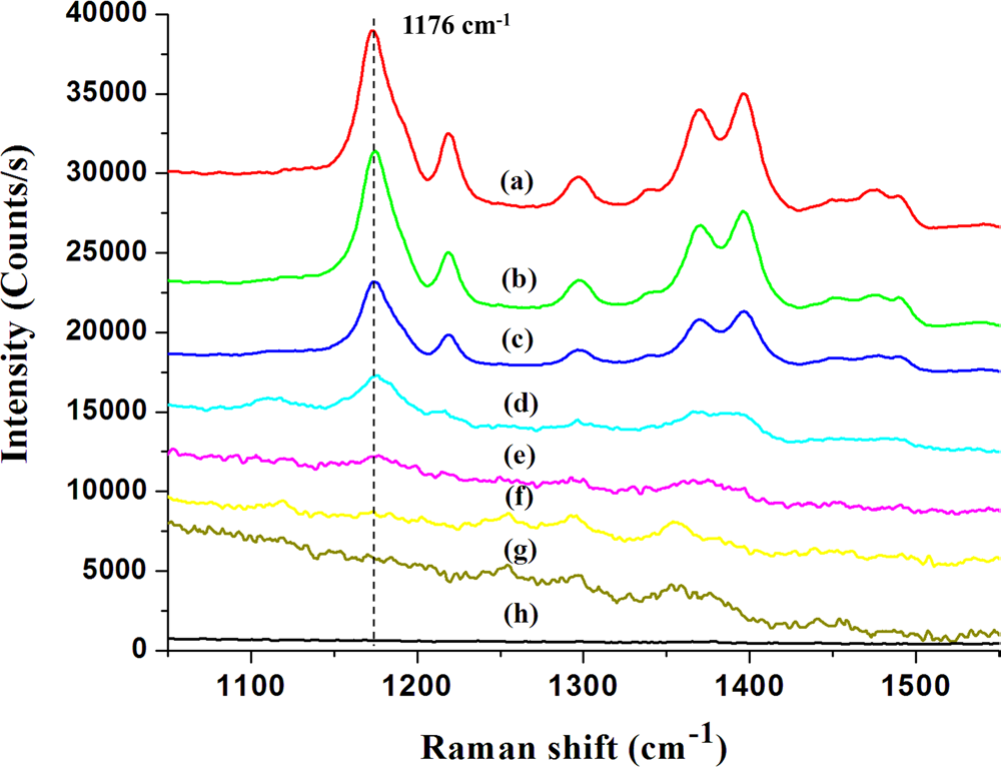
Raman spectrums of MG with various concentrations measured by optimized SERS substrate. The labeled represented different concentrations of MG as following (**a**) 10 mM, (**b**) 1 mM, (**c**) 100 μM, (**d**) 10 μM, (**e**) 1 μM, (**f**) 100 nM, (**g**) 10 nM, (**h**) Blank (SERS substrate without MG).

**Figure 5:**
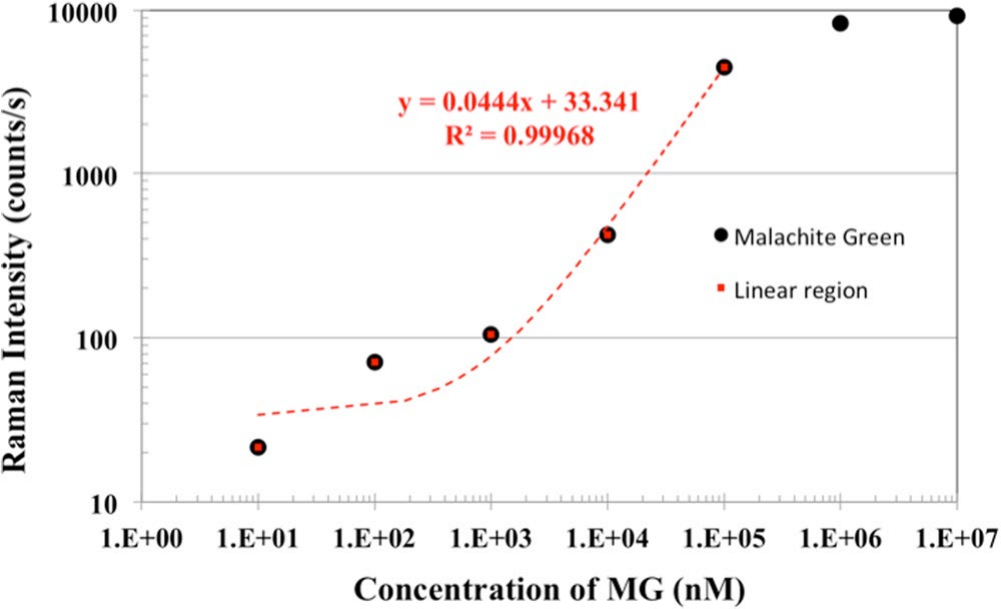
The black symbols represented the relationship (logarithm scale) of peak intensity (sited around 1176 cm^−1^) and various concentration of MG measured by the optimized SERS substrate. The red symbols represented the linear region of quantitative measurement. It is noted that the laser setup is the same as previous Raman measurement.

Our preliminary results demonstrated an optimized highly sensitivity SERS substrate by applying low cost process and statistical experiment design method. The optimized substrate possessed well stability and suitable for practical analysis. In practical analysis, the quantitative measurement is hard to achieve because of the strong variation of substrate. Here, we demonstrated a practical application of detecting malachite green (MG). MG is a cheap and powerful drug that has been widely used in the aquaculture industry to prevent fishes from the fungal and parasite infections. However, the residue of MG will cause carcinogenic risk to humans. In Ireland, the concentration of MG in fish farm effluent was limited under 100 μg/L (corresponding to about 274 nM)^28^. In this work, the optimized SERS substrate composed of SiNWA decorated with AuNPs and AuMBP is applied to detect various concentrations of MG. The MG solutions were prepared and diluted to several concentrations ranging from 10 mM to 10 nM. The pipette was used to extract 3 μL of each concentration of MG solutions. Then, the 3 μL MG solutions were dropped onto the optimized SERS substrate and dried under normal atmosphere environment. In Fig. 4, the measured Raman spectra correspond to various concentrations of MG. By the optimized SERS substrates, the detection limit equal to 10 nM was achieved. Furthermore, we selected the strongest characteristic peak sited at 1176 cm^−1^, which corresponds to the in-plane vibrations of ring C-H, to describe the relationship between the peak intensity and the concentration of MG. It is noted that the blank line in Fig. 4(h) shows the clean background of our developed SERS substrate. As shown in Fig. 5, the intensities of Raman peaks increased monotonously with greater concentration of MG and finally saturated with concentration higher than 100 μM. The saturated phenomena were due to the surface area of SERS-active region covered by the MG molecules. In particular, the strong linear dependence of Raman intensity between 10 nM to 100 μM provided a great relation for quantitatively detection shown as red regression line in Fig. 5.

In conclusion, we demonstrated a facile method to fabricate a low-cost, high-sensitivity SERS substrate consisting of SiNWA decorated with both AuNPs and AuMBP. Based on a statistical experimental design by Taguchi method, we further optimized this kind of nanostructured SERS substrates in an efficient manner. By employing the optimized SERS substrate to the Raman measurement of thiophenol molecule, the average Raman signal soared up to 1740 counts/s, which was 1.78 times higher than the commercialized SERS substrate. When using the commercialized Klarite SERS substrate with enhancement factor near 10^6^ ^29^ as the benchmark, our optimized SERS substrate showed an enhancement factor of 1.78 × 10^6^. Moreover, it equipped a low coefficient of variance (CV) of 4.2%. Finally, in the real world application, the detection limit of residue MG concentration was equal to 10 nM, which met the demand of MG residue detection in fish farm water. Moreover, the results exhibited a strong linear dependence (R^2^ = 0.999) from 10 nM to 100 μM for MG quantitative analysis. In short, such a low-cost, high-sensitivity SERS substrate substantially outperformed the commercialized SERS substrate, and promised a rapid and accurate screening for both qualitative and quantitative analysis of toxic residue trace detection.

## Electronic supplementary material

Supplementary information
