# Supplementary material for: A Low-cost, Highly-stable Surface Enhanced Raman Scattering Substrate by Si Nanowire Arrays Decorated with Au Nanoparticles and Au Backplate
Source: Sci Rep. 2017 Jul 4;7:4604. doi: 10.1038/s41598-017-04062-4 (PMC5496898; doi:10.1038/s41598-017-04062-4)
Supplement: Supplementary file 1 — Supplementary information [file 41598_2017_4062_MOESM1_ESM.docx]

**Supplementary Material**

**A Low-cost, Highly-stable Surface Enhanced Raman Scattering Substrate by Si Nanowire Arrays Decorated with Au Nanoparticles and Au Backplate**

Bi-Shen Lee(李璧伸),^1^ Ding-Zheng Lin (林鼎晸),^2^  and Ta-Jen Yen (嚴大任)^1^,*

^1^Department of Material Science and Engineering, National Tsing Hua University, Hsinchu 30013, Taiwan

^2^Department of Material and Chemical Research Laboratories, Industrial technology and research institute (ITRI), Hsinchu, Taiwan

Email: [**^*^**tjyen@mx.nthu.edu.tw](mailto:*tjyen@mx.nthu.edu.tw)

**Details of the Taguchi analysis:**

To obtain the optimized SERS substrate with the greatest Raman signal intensity, the

S/N (signal-to-noise) ratio related to the larger the better (TLTB) quality characteristics were conducted. For TLTB quality characteristics, the S/N ratio was given by the following equation:

S/N ratio (η) = $-10\log\left(\frac{\sum_{i=1}^{n} (1/y{}_{i}^{2})}{n}\right)$

In which n and y_i_ represented the number of values and the Raman signal intensity, respectively. We conducted an analysis of variance (ANOVA) to examine the influence of factors on the quality characteristics. The calculated values of S/N ratio for each trial were presented in Table S1 and the S/N ratio values were conducted to determine the optimal settings of control factors in Figure S1. According to the criteria of S/N ratio, the optimal levels of each process parameter were determined as A2B2C3D3, which denoted 300nm-long SiNWA with 70° OAD, 30nm-thick AuNPs and 20nm-thick AuMBP, respectively.

Table S1. The Taguchi L9 orthogonal array and its results of S/N ratio for each trial

| Trial No. |  | Control factor level | | | | Signal to noise (S/N) ratio |
| --- | --- | --- | --- | --- | --- | --- |
|  |  | A. Depths of SiNWA (nm) | B. OAD angle (degree) | C. Thickness of AuNPs (nm) | D. Thickness of AuMBP (nm) |  |
| 1 |  | 150 | 60 | 10 | 0 | 47.29 |
| 2 |  | 150 | 70 | 20 | 10 | 56.35 |
| 3 |  | 150 | 80 | 30 | 20 | 59.22 |
| 4 |  | 300 | 60 | 20 | 20 | 56.95 |
| 5 |  | 300 | 70 | 30 | 0 | 55.28 |
| 6 |  | 300 | 80 | 10 | 10 | 59.14 |
| 7 |  | 600 | 60 | 30 | 10 | 58.88 |
| 8 |  | 600 | 70 | 10 | 20 | 59.41 |
| 9 |  | 600 | 80 | 20 | 0 | 51.10 |


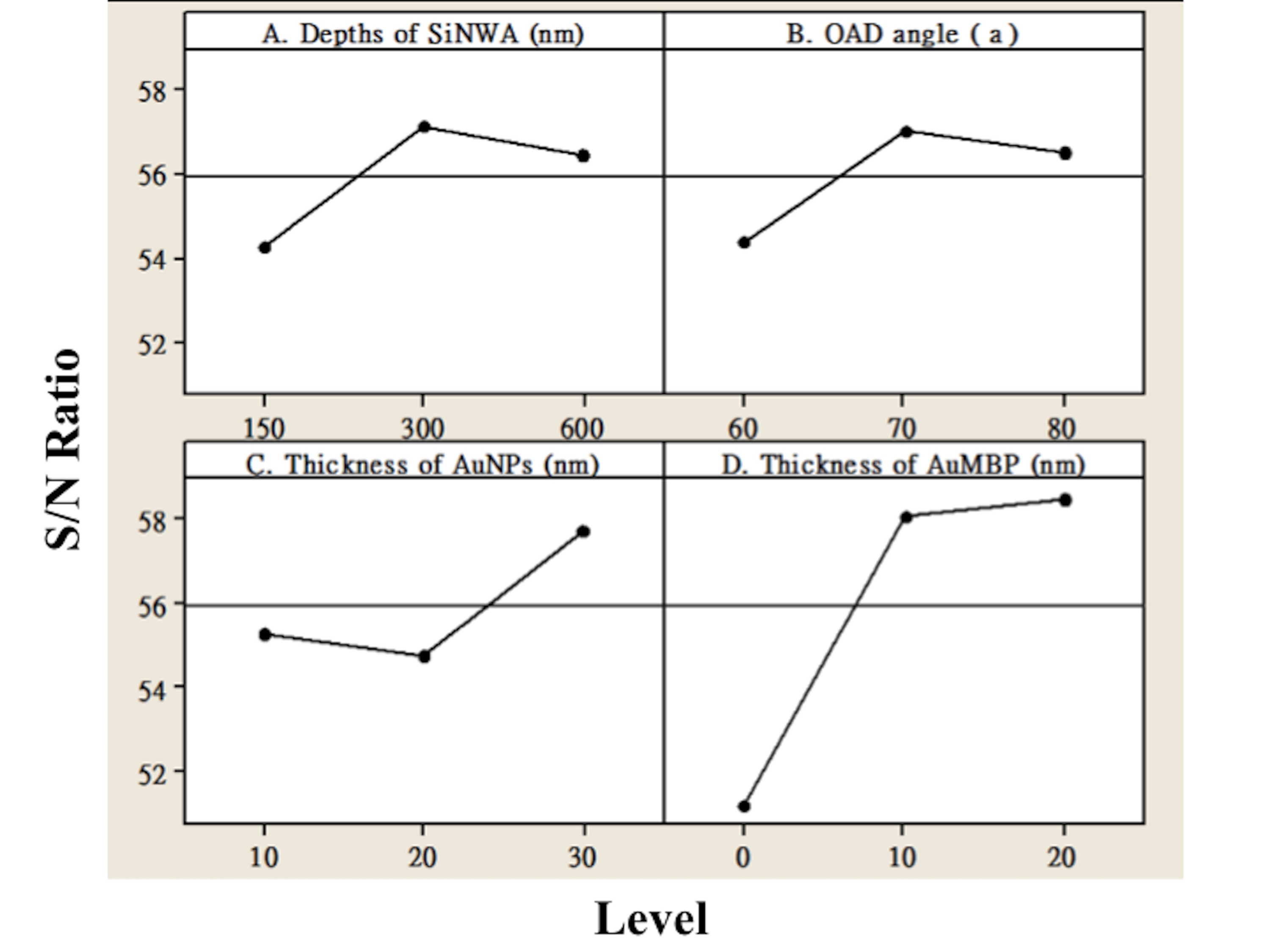


Figure S1. The results of ANOVA analysis and S/N ratio of different parameter levels indicated the optimized combination. (The optimized parameter combination was A_2_B_2_C_3_D_3_, which denoted 300nm-long SiNWA with 70° OAD, 30nm-thick AuNPs and 20nm-thick AuMBP, respectively)
